# Stereotactic Body and Conventional Radiotherapy for Painful Bone Metastases: A Systematic Review and Meta-Analysis

**DOI:** 10.1001/jamanetworkopen.2023.55409

**Published:** 2024-02-12

**Authors:** Bas J. J. Bindels, Carole Mercier, Roxanne Gal, Jorrit-Jan Verlaan, Joost J. C. Verhoeff, Piet Dirix, Piet Ost, Nicolien Kasperts, Yvette M. van der Linden, Helena M. Verkooijen, Joanne M. van der Velden

**Affiliations:** 1 Department of Orthopedic Surgery, University Medical Center Utrecht, Utrecht, the Netherlands; 2 Department of Radiation Oncology, Iridium Netwerk, Antwerpen, Belgium; 3 Integrated Personalised and Precision Oncology Network, University Antwerp, Antwerp, Belgium; 4 Division of Imaging and Oncology, University Medical Center Utrecht, Utrecht University, the Netherlands; 5 Department of Radiation Oncology, University Medical Center Utrecht, Utrecht, the Netherlands; 6 Department of Human Structure and Repair, Ghent University, Ghent, Belgium; 7 Department of Radiation Oncology and Centre of Expertise in Palliative Care, Leiden University Medical Center, Leiden, the Netherlands; 8 Netherlands Comprehensive Cancer Organization, Utrecht, the Netherlands; 9 Julius Center for Health Sciences and Primary Care, University Medical Center Utrecht, Utrecht, the Netherlands

## Abstract

**Importance:**

Conventional external beam radiotherapy (cEBRT) and stereotactic body radiotherapy (SBRT) are commonly used treatment options for relieving metastatic bone pain. The effectiveness of SBRT compared with cEBRT in pain relief has been a subject of debate, and conflicting results have been reported.

**Objective:**

To compare the effectiveness associated with SBRT vs cEBRT for relieving metastatic bone pain.

**Data Sources:**

A structured search was performed in the PubMed, Embase, and Cochrane databases on June 5, 2023. Additionally, results were added from a new randomized clinical trial (RCT) and additional unpublished data from an already published RCT.

**Study Selection:**

Comparative studies reporting pain response after SBRT vs cEBRT in patients with painful bone metastases.

**Data Extraction and Synthesis:**

Two independent reviewers extracted data from eligible studies. Data were extracted for the intention-to-treat (ITT) and per-protocol (PP) populations. The study is reported following the Preferred Reporting Items for Systematic Reviews and Meta-analyses (PRISMA) reporting guideline.

**Main Outcomes and Measures:**

Overall and complete pain response at 1, 3, and 6 months after radiotherapy, according to the study’s definition. Relative risk ratios (RRs) with 95% CIs were calculated for each study. A random-effects model using a restricted maximum likelihood estimator was applied for meta-analysis.

**Results:**

There were 18 studies with 1685 patients included in the systematic review and 8 RCTs with 1090 patients were included in the meta-analysis. In 7 RCTs, overall pain response was defined according to the International Consensus on Palliative Radiotherapy Endpoints in clinical trials (ICPRE). The complete pain response was reported in 6 RCTs, all defined according to the ICPRE. The ITT meta-analyses showed that the overall pain response rates did not differ between cEBRT and SBRT at 1 (RR, 1.14; 95% CI, 0.99-1.30), 3 (RR, 1.19; 95% CI, 0.96-1.47), or 6 (RR, 1.22; 95% CI, 0.96-1.54) months. However, SBRT was associated with a higher complete pain response at 1 (RR, 1.43; 95% CI, 1.02-2.01), 3 (RR, 1.80; 95% CI, 1.16-2.78), and 6 (RR, 2.47; 95% CI, 1.24-4.91) months after radiotherapy. The PP meta-analyses showed comparable results.

**Conclusions and Relevance:**

In this systematic review and meta-analysis, patients with painful bone metastases experienced similar overall pain response after SBRT compared with cEBRT. More patients had complete pain alleviation after SBRT, suggesting that selected subgroups will benefit from SBRT.

## Key Points

**Question:**

Is stereotactic body radiotherapy (SBRT) associated with superior relief of metastatic bone pain compared with conventional external beam radiotherapy (cEBRT)?

**Findings:**

In this systematic review and meta-analyses of 8 randomized clinical trials and 1090 patients, the overall pain response did not differ between patients treated with cEBRT and SBRT after 1, 3, or 6 months. More patients experienced complete pain alleviation after SBRT than after cEBRT at all 3 time points.

**Meaning:**

This systematic review and meta-analysis does not support the routine use of SBRT for all patients with painful bone metastases, but selected subgroups may benefit from SBRT.

## Teaser

This systematic review and meta-analysis assesses the comparative effectiveness associated with stereotactic body radiotherapy vs conventional external beam radiotherapy for relief of metastatic bone pain.

## Introduction

Bone metastases may cause severe pain^1^ and substantially reduce quality of life.^2^ Conventional external beam radiotherapy (cEBRT) and stereotactic body radiotherapy (SBRT) are effective treatment modalities for relieving metastatic bone pain.^3^ Compared with cEBRT, SBRT allows higher doses to the target area while sparing surrounding tissues and nearby organs at risk. Higher doses may further improve pain response in patients with metastatic bone pain.^4,5^

In 2019, Spencer et al^6^ reviewed SBRT effectiveness, finding superior pain response and lower toxic effects rates compared with cEBRT. However, most studies were nonrandomized, introducing selection bias. Given the methodological limitations of the available literature at the time, large randomized clinical trials (RCTs) were needed.

Since 2019, conflicting results from RCTs and comparative studies on pain response have been published.^7,8,9,10,11,12,13^ To aggregate the results of these newer studies, several meta-analyses assessed pain response for metastatic bone disease and again published conflicting conclusions.^14,15,16,17,18^ In our review, we included the largest RCT^19^ to our knowledge and an eighth RCT on this subject.^20^ Additionally, we assessed unpublished results from an RCT previously conducted by our team.^8^ Using these data, we conducted a systematic review and meta-analysis with the updated trial data to evaluate the comparative effectiveness associated with SBRT vs cEBRT for relieving metastatic bone pain.

## Methods

This systematic review and meta-analysis was conducted following the updated guidelines of the Preferred Reporting Items for Systematic Reviews and Meta-analyses (PRISMA) reporting guideline.^21^ The study protocol was registered in PROSPERO (CRD42021264315).^22^

### Search Strategy

A structured search was developed with a licensed librarian and last updated on June 5, 2023. The search aimed to identify comparative studies reporting pain response in patients with painful bone metastases after SBRT or cEBRT. The PubMed, Embase, and Cochrane electronic databases were searched using the search terms *bone metastases* and *stereotactic body radiotherapy* and synonyms, which were combined and searched in title and abstract (eTable 1 in Supplement 1). Study protocols were followed up and reference lists from included articles were cross-checked to identify other potential articles. We also included the full results from a recently completed RCT^20^ and unpublished data from an already published RCT^8^ through collaboration with the investigators.

### Study Selection

After removing duplicates, 2 authors (B.J.J.B. and J.M.V.D.V.) independently assessed studies for eligibility. All comparative studies assessing pain response in patients with bone metastases from solid tumors who underwent cEBRT or SBRT were included. Pain response had to be reported on a patient level. Studies including patients who had received previous radiotherapy or surgery at the target site were excluded. We also excluded studies not written in English or those not presenting original research. When individual patients were reported in multiple published studies, the most complete or recent article was included.^23^ Full texts were reviewed if eligibility could not be determined based on title and abstract. Any disagreements were resolved by consensus. Screening of the studies was facilitated by systematic review software (Rayyan).^24^

### Data Extraction and Quality Assessment

The primary outcome was overall pain response. Secondary outcomes included complete pain response, local tumor control and progression-free survival, toxic effects, pathological fractures, quality of life, and overall survival.

Definition of pain response was derived according to the definition of the original study. Pain response was expressed as the proportion of patients experiencing pain response at a certain point in time. If available, the proportion of responders was recorded or calculated for the intention-to-treat (ITT) population (ie, patients who were assigned to the intended treatment) and for the per-protocol (PP) population (ie, patients who received the intended treatment). Pain response was recorded 1, 3, 6, 9, and 12 months after treatment, if reported. Toxic effects were collected if scored according to the Common Terminology Criteria for Adverse Events versions 3.0 to 6.0. Pathological fractures were defined as (progression of) any fracture occurring at the irradiated site. For each study, the biologically effective dose (BED_10_) and the equivalent dose delivered in 2 Gy (EQD2) were calculated for the regimens applied. We assumed an α:β ratio of 10 to calculate the EQD2 and BED_10_. The BED_10_ and EQD2 are measures to compare different treatment regimens.

Study and patient characteristics were extracted independently by 2 authors (B.J.J.B. and J.M.V.D.V.). The methodological quality for RCTs was critically appraised using the Cochrane revised tool for assessing risk of bias,^25^ and for nonrandomized studies using predefined criteria based on the Strengthening the Reporting of Observational Studies in Epidemiology (STROBE) reporting guideline for reporting observational studies.^6,26^

### Statistical Analysis

Pain response, a dichotomous end point, was expressed as risk ratio (RR) with 95% CI. Random-effects models, using a restricted maximum likelihood estimator, were used to calculate a pooled estimate regardless of the *I*^2^ measure of heterogeneity. In addition, for SBRT and cEBRT separately, the pain response was pooled to calculate a pooled proportion using the raw proportions. The pooled proportions are presented with 95% CIs. Random-effects models and pooled proportions were calculated for pain response at 1, 3, and 6 months after radiotherapy. Studies included in the random-effects models were ordered based on the highest calculated EQD2 for SBRT, and we visually assessed whether the EQD2 was associated with pain response. Outcomes not amenable to meta-analytic pooling because of inconsistent definitions or measurement methods were summarized. Potential publication bias was visually assessed with funnel plots.^27^ Analyses were performed using R software version 4.0.3 (R Project for Statistical Computing) metafor package version 4.2-0. *P* values were 2-sided, and *P* = .05 was considered as significant. Data were analyzed from June 5 to August 15, 2023.

## Results

The search yielded 8284 unique articles. After title and abstract screening, 92 studies needed full-text screening, of which 17 studies^7,8,9,10,11,12,13,19,28,29,30,31,32,33,34,35,36^ were included in the review. Additionally, we included 1 recently completed RCT^20^ and the unpublished complete pain response data from an already published RCT^8^ (eFigure 1 in Supplement 1). Finally, 18 comparative studies^7,8,9,10,11,12,13,19,20,28,29,30,31,32,33,34,35,36^ were included in the review, with 1685 patients. Of these 18 comparative studies^7,8,9,10,11,12,13,19,20,28,29,30,31,32,33,34,35,36^, 3 studies^34,35,36^ published secondary outcomes from 2 included RCTs^8,33^ reporting on pain response. The funnel plots showed some asymmetry, suggesting limited publication bias (eFigure 2 in Supplement 1).

### Quality Assessment

Eight RCTs,^7,8,9,10,19,20,29,33^ 1 prospective study,^13^ and 6 retrospective cohort studies^11,12,28,30,31,32^ reported pain responses at 1, 3, 6, 9, and/or 12 months after radiotherapy. The RCTs were considered to have a low risk of bias or with some methodological concerns, except for the study of Sakr et al,^10^ which was considered to be at high risk of bias. A sensitivity analysis excluding this study from the meta-analyses did not change the study findings (eFigure 7 in Supplement 1). All observational studies had a high risk of bias concerning the comparability of study groups or the moment of outcome assessment (eFigure 3 in Supplement 1). Therefore, we decided only to include the RCTs in the meta-analysis.

### Study Description

Between 2010 and 2022, the 8 phase 2 or 3 RCTs^7,8,9,10,19,20,29,33^ randomized 1090 patients, of whom 980 (90%) underwent their allocated treatment (462 patients underwent cEBRT [47%] and 518 patients underwent SBRT [53%]). The 2 phase 3 RCTs^9,19^ included 582 patients. Three RCTs^9,19,33^ only included spinal lesions, and 5 RCTs^7,8,10,20,29^ included both spinal and nonspinal lesions. Lung cancer was the most prevalent primary tumor in 6 RCTs,^7,8,9,20,29,33^ prostate cancer in 1 RCT,^10^ and 1 RCT^19^ did not report the prevalence of primary tumors. Most RCTs^7,8,10,19,29^ reported that most patients had a baseline pain score of 6 or higher (on a scale from 0 to 10) (Table 1).

**Table 1.** Study Characteristics of the 15 Included Studies

Source	Study design	Years of treatment	Total ITT population, No./total PP population, No.	3 Most prevalent primary tumors (%)	Age, cEBRT /SBRT, y	Performance status, cEBRT/SBRT, %	Locations of irradiated bone lesions	Pain score at baseline, cEBRT/SBRT	Regimen, Gy/Fx		EQD2 or BED_10_, Gy^a^	
									cEBRT	SBRT	cEBRT	SBRT
**RCT**												
Mercier et al,^20^ 2023	Phase 3 RCT	2019-2022	126/123	Lung (31.7); prostate (23.8); breast (15.9)	67/68 (median)	ECOG 0-1 82.5/79.4	All bones	NRS 8-10: 14.2%/30.2%	8/1	20/1	12.0/14.4	50.0/60.0
Sakr et al,^10^ 2020	Phase 2 RCT	2018-2019	22/22	Prostate (18.2); HCC (18.2); sarcoma (13.6)	58/58 (median)	NR	All bones	NRS: 6.0/8.0 (median)	20/5	27/3	23.3/28.0	42.8/51.3
Sahgal et al,^9^ 2021	Phase 3 RCT	2016-2019	229/223	Lung (26.6); breast (21.8); GU (20.1)	65/63 (median)	ECOG 0-1: 90.4/93.0	C/T/L/S spine	NRS: 5.0/5.0 (median)	20/4, 20/5	24/2	25.0/30.0 or 23.3/28	44.0/52.8
Pielkenrood et al,^8^ 2021	Phase 2 RCT	2015-2019	110/70	Lung (25.8); prostate (22.5); breast (19.1)	63/65 (median)	KPS 80-100: 40.9/42.2	All bones	NRS: 6.2/6.6 (median)	8/1; 20/5; 30/10	18/1; 30/3; 35/5	12.0/14.4^b^	50.0/60.0^b^
Nguyen et al,^7^ 2019	Phase 2 RCT	2014-2018	160/133	Lung (49.3); prostate (14.3); breast/RCC (8.8)	63/62 (mean)	KPS 70-80: 73.4/70.4	All bones	NRS 7-10: 62.0%/44.4%	30/10	12/1; 16/1	32.5/39.0	22.0/26.4 or 34.7/41.6
Sprave et al,^33^ 2018	Phase 2 RCT	2014-2017	60/55	Lung (34.5); breast (30.9); RCC (7.3)	64/61 (mean)	Only patients with KPS>70	T/L spine	VAS: 4.6/3.9 (mean)	30/10	24/1	32.5/39.0	68.0/81.6
Ryu et al,^19^ 2023	Phase 3 RCT	2011-2017	353/302	NR	63/62 (mean)	Zubrod 0-1: 90.0/78.0	C/T/L spine	NRS: 7/7 (median)	8/1	16/1; 18/1	12.0/14.4	34.7/41.6 or 42.0/50.4
Berwouts et al,^29^ 2015	Phase 2 RCT	2010-2014	30/25	Lung (43.3); prostate (16.7); breast (16.7)	63/63 (mean)	KPS 70-80: 46.7/33.3	All bones	NRS 7-10: 60.0%/33.3%	8/1	16/1	12.0/14.4	34.7/41.6
**Cohort studies**												
Ito et al,^11^ 2022	RCS	2013-2022	NA/162	Lung (36.4); prostate (11.1); RCC (11.1)	67/68 (median)	ECOG 0-1: 82.7/85.2	Nonspine bones	NRS 8-10 28.4%/29.6%	8/1; 20/5; 30/10	24/2; 30/5; 35/5	32.5/39.0^c^	49.6/59.5^c^
Marvaso et al,^12^ 2022	RCS	2015-2020	NA/121	Lung (100)	NR	NR	All bones	NR	NR	NR	NR	NR
Van de Ven et al,^13^ 2020	PCS	2013-2017	NA/131	Prostate (29.8); breast (23.7); lung (22.1)	68/64 (mean)	ECOG 0-1: 62.1/64.6	All bones	NRS: 4.6/3.0 (mean)	8/1; 20/5; 30/10	18/1; 30/3; 35/5	12.0/14.4^c^	42.0/50.4^c^
Amini et al,^28^ 2015	RCS	2004-2014	NA/95	RCC (100)	62 (median)	NR	All bones	NR	8-10/1; 20/5; 24/8; 30-40/10-12	12-20/1; 21-35/3; 25-50/5	23.3/28.0^c^	42.8/51.3^c^
Sohn et al,^32^ 2016	RCS	2005-2012	NA/56	HCC (100)	60/59 (mean)	ECOG 0-1: 78.5/67.9	C/T/L spine	VAS: 5.6/6.8 (mean)	32 (mean)/10 (mean)	35 (mean)/1-5	NR/33.7 (mean)^d^	NR/58.4 (mean)^d^
Sohn et al,^31^ 2014	RCS	2005-2012	NA/26	RCC (100)	61/62 (mean)	ECOG 0-1: 53.8/76.9	C/T/L/S spine	VAS: 6.0/7.3 (mean)	29 (mean)/11 (median)	38 (mean)/1-5	NR/32 (mean)^d^	NR/61.7 (mean)^d^
Haley et al,^30^ 2011	RCS	NR	NA/44	Breast (50.0); lung (36.0); renal (9.0)	57/56 (median)	NR	C/T/L spine	NR	20-35/5-10	14-20/1	23.3/28.0^c^	34.7/41.6^c^

Abbreviations: BED_10_, biologically effective dose; C, cervical; cEBRT; conventional external beam radiotherapy; ECOG, Eastern Cooperative Oncology Group; EQD2, equivalent dose delivered in 2 Gy; Fx, fractions; GU, genitourinary; HCC, hepatocellular carcinoma; ITT, intention-to-treat; KPS, Karnofsky Performance Score; L, lumbar; NA, not applicable; NR, not reported; NRS, numerical rating scale; PCS, prospective cohort study; PP, per-protocol; RCC, renal cell carcinoma; RCS, retrospective cohort study; RCT, randomized clinical trial; S, sacral; SBRT, stereotactic body radiotherapy; T, thoracic; VAS, visual analog scale.

^a^ An α:β ratio of 10 was assumed.

^b^ Based on 8 Gy in 1 Fx and 30 Gy in 3 Fx, which were the most prevalent regimen (data retrieved from unpublished data set).

^c^ Based the most prevalent regimen in the study.

^d^ The EQD2 and BED_10_ could not be calculated for this study, but the mean BED_10_ was reported (based on an α:β ratio of 10).

### Pain Response

Pain response was mostly reported 1, 3, and 6 months after radiotherapy. In 7 of 8 RCTs,^7,8,9,10,20,29,33^ pain response was defined according to the International Consensus on Palliative Radiotherapy Endpoints in clinical trials (ICPRE).^37^ The ICPRE considers pain response a partial or complete response determined on an 11-point scale. Partial pain response is defined as a decline of at least 2 points without an increase in opioid use. Complete pain response is defined as a pain score of 0 without an increase in opioid use. In 2 RCTs,^9,20^ the primary end point was complete pain response according to the ICPRE, but the trials also reported the number of patients experiencing partial pain response. Ryu et al^19^ defined pain response as a 3-point decrease in pain score on a scale of 10 points (Table 2). From the RCTs defining pain response according to the ICPRE, we used both complete and partial pain responders for our meta-analysis on overall pain response and from Ryu et al,^19^ we used the pain responders according to their definition.

**Table 2.** Overview of Overall Pain Response in the Intention-to-Treat and Per-Protocol Populations From the 15 Included Studies

Source	Study design	Definition of PR	ITT or PP	Patients with PR, No./ total patients, No. (%)							
				Month 1		Month 3		Month 6		Month 9-12	
				cEBRT	SBRT	cEBRT	SBRT	cEBRT	SBRT	cEBRT	SBRT
**RCTs**											
Mercier et al,^20^ 2023	RCT 3	Consensus^a^	ITT	39/63 (61.9)	44/63 (69.8)	28/63 (44.4)	32/63 (50.8)	NA	NA	NA	NA
			PP	39/63 (61.9)	43/60 (71.7)	28/48 (58.3)	32/39 (82.1)	NA	NA	NA	NA
Sakr et al,^10^ 2020	RCT 2	Consensus^a^	ITT	NA	NA	9/12 (75.0)	8/10 (80.0)	NA	NA	NA	NA
			PP	NA	NA	9/12 (75.0)	8/10 (80.0)	NA	NA	NA	NA
Sahgal et al,^9^ 2021	RCT 3	Consensus ^a^	ITT	53/115 (46.1)	64/114 (56.1)	45/115 (39.1)	60/114 (52.6)	36/115 (31.3)	47/114 (41.2)	NA	NA
			PP	53/105 (50.5)	64/99 (64.6)	45/93 (48.3)	60/94 (63.8)	36/76 (47.4)	47/78 (60.3)	NA	NA
Pielkenrood et al,^8^ 2021	RCT 2	Consensus^a^	ITT	19/55 (34.5)	16/55 (29.1)	14/55 (31.8)	18/55 (32.7)	NA	NA	NA	NA
			PP	19/44 (43.2)	10/26 (38.5)	14/44 (31.8)	12/26 (46.2)	NA	NA	NA	NA
Nguyen et al,^7^ 2019	RCT 2	Consensus^a^	ITT	24/79 (30.4)	36/81 (44.4)	17/79 (21.5)	31/81 (38.3)	17/79 (21.5)	19/81 (23.5)	12/79 (15.2)	17/81 (21.0)
			PP	24/44 (54.5)	36/44 (81.8)	17/35 (48.6)	31/43 (72.1)	17/28 (60.7)	19/28 (67.9)	12/26 (46.2)	17/22 (77.3)
Sprave et al,^33^ 2018	RCT 2	Consensus^a^	ITT	NA	NA	11/30 (36.7)	16/30 (39.3)	7/30 (23.3)	14/30 (46.7)	NA	NA
			PP	NA	NA	11/23 (47.8)	16/23 (69.6)	7/20 (35.0)	14/19 (73.7)	NA	NA
Ryu et al,^19^ 2023	RCT 2	Own definition	ITT	44/136 (32.4)	70/217 (32.3)	46/136 (33.8)	57/217 (26.3)	25/136 (18.4)	38/217 (17.5)	21/136 (15.4)	33/217 (15.2)
			PP	44/93 (47.3)	70/153 (45.8)	46/76 (60.5)	57/138 (41.3)	25/39 (64.1)	38/68 (55.9)	21/38 (55.3)	34/59 (57.6)
Berwouts et al,^29^ 2015	RCT 2	Consensus^a^	ITT	8/15 (53.3)	9/15 (60.0)	NA	NA	NA	NA	NA	NA
			PP	8/12 (66.7)	9/13 (69.2)	NA	NA	NA	NA	NA	NA
**Cohort studies**											
Ito et al,^11^ 2022	RCS	Consensus^a^	ITT	NR	NR	NR	NR	NR	NR	NA	NA
			PP	45/73 (61.6)	58/75 (77.3)	46/81 (56.8)	62/81 (76.5)	21/42 (50.0)	44/58 (75.9)	NA	NA
Marvaso et al,^12^ 2022	RCS	Consensus^a^	ITT	NA	NA	NR	NR	NA	NA	NA	NA
			PP	NA	NA	15/59 (25.4)^b^	22/62 (35.5)^b^	NA	NA	NA	NA
Van de Ven et al,^13^ 2020	PCS	Consensus^a^	ITT	NA	NA	NR	NR	NR	NR	NR	NR
			PP	NA	NA	27/40 (67.5)	18/35 (51.4)	9/34 (55.9)	18/28 (64.3)	13/26 (50.0)	8/10 (80.0)
Amini et al,^28^ 2015	RCS	Own definition	ITT	NA	NA	NA	NA	NA	NA	NR	NR
			PP	NA	NA	NA	NA	NA	NA	NR (39.9)	NR (74.9)
Sohn et al,^32^ 2016	RCS	Consensus^a^	ITT	NR	NR	NA	NA	NA	NA	NA	NA
			PP	16/28 (57.1)	18/28 (64.3)	NA	NA	NA	NA	NA	NA
Sohn et al,^31^ 2014	RCS	Consensus^a^	ITT	NR	NR	NA	NA	NA	NA	NA	NA
			PP	6/13 (46.2)	10/13 (76.9)	NA	NA	NA	NA	NA	NA
Haley et al,^30^ 2011	RCS	Own definition	ITT	NR	NR	NA	NA	NA	NA	NA	NA
			PP	NR	NR	NA	NA	NA	NA	NA	NA

Abbreviations: cEBRT, conventional external beam radiotherapy; ITT, intention-to-treat; NA, not applicable; NR, not reported; PCS, prospective cohort study; PP, per-protocol; PR, pain response; RCS, retrospective cohort study; RCT, randomized controlled trial; SBRT, stereotactic body radiotherapy.

^a^ International Consensus on Palliative Radiotherapy Endpoints in clinical trials.

^b^ Only complete pain response according to International Consensus.

**Figure 1.**
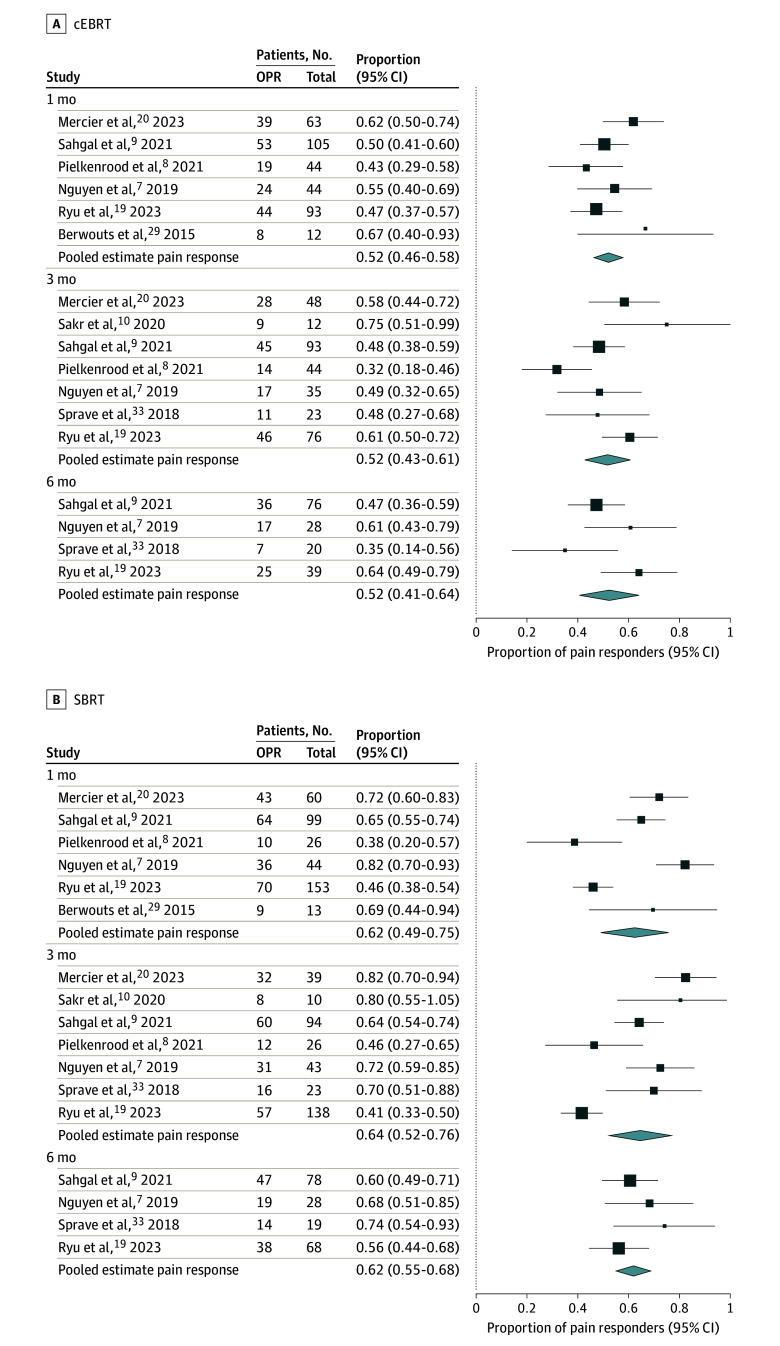
Pooled Overall Pain Response (OPR) Among the Per-Protocol Population of the 8 Included Randomized Clinical Trials at 1, 3, and 6 Months The trials compared conventional external beam radiotherapy (cEBRT) with stereotactic body radiotherapy (SBRT).

In the PP population, the pooled overall pain response rates after cEBRT were 52% (95% CI, 46%-58%) after 1 month, 52% (95% CI, 43%-61%) after 3 months, and 52% (95% CI, 41%-64%) after 6 months. After SBRT, the pooled overall response rates in the PP population were 62% (95% CI, 49%-75%) after 1 month, 64% (95% CI, 52%-76%) after 3 months, and 62% (95% CI, 55%-68%) after 6 months. The 95% CIs of the pooled overall pain response rates overlapped at each time point (Figure 1). The pooled complete pain response rates after cEBRT were 18% (95% CI, 12%-24%) after 1 month, 16% (95% CI, 7%-24%) after 3 months, and 18% (95% CI, 4%-31%) after 6 months. After SBRT, the pooled complete response rates were 26% (95% CI, 14%-38%) after 1 month, 31% (95% CI, 12%50%) after 3 months, and 48% (95% CI, 39%-58%) after 6 months (eFigure 4 in Supplement 1).

**Figure 2.**
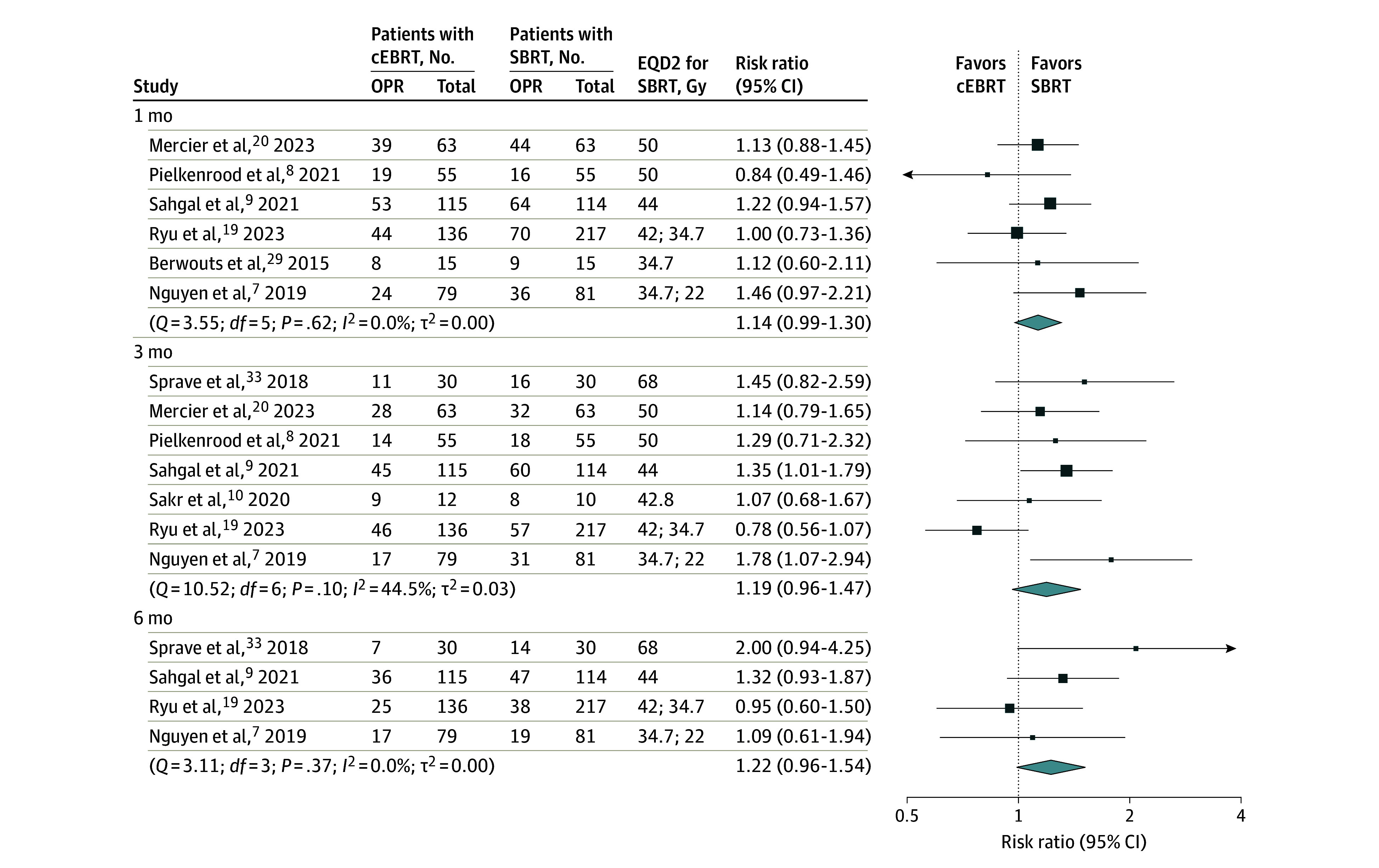
Intention-to-Treat Meta-Analysis on Overall Pain Response (OPR) at 1, 3, and 6 Months After Radiotherapy of the 8 Included Randomized Trials The trials compared conventional external beam radiotherapy (cEBRT) with stereotactic body radiotherapy (SBRT). Studies were sorted based on the equivalent dose delivered in 2 Gy (EQD2) for SBRT, with the highest dose on top.

In the ITT meta-analysis, the pooled overall pain response did not differ between SBRT and cEBRT after 1 (RR, 1.14; 95% CI, 0.99-1.30), 3 (RR, 1.19; 95% CI, 0.96-1.47), or 6 (RR, 1.22; 95% CI, 0.96-1.54) months (Figure 2). In the PP meta-analysis, SBRT was associated with a higher pain response than cEBRT at 1 month (RR, 1.17; 95% CI, 1.01-1.36) (eFigure 5 in Supplement 1). No association was seen between SBRT EQD2 and pain response.

**Figure 3.**
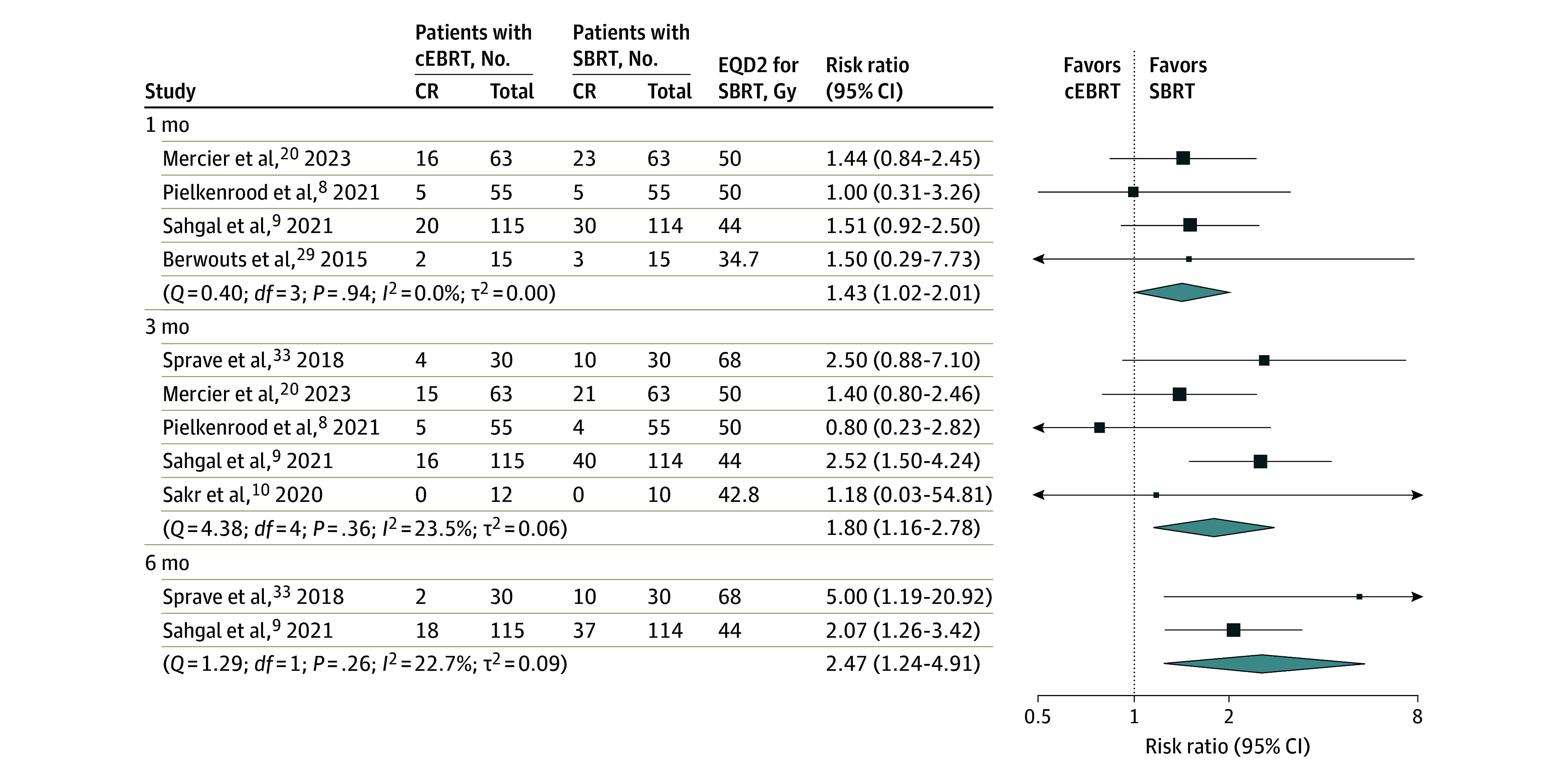
Intention-to-Treat Meta-Analysis on Complete Pain Response (CR) at 1, 3, and 6 Months After Radiotherapy of the 6 Included Randomized Trials The trials compared conventional external beam radiotherapy (cEBRT) with stereotactic body radiotherapy (SBRT). Studies were sorted based on the equivalent dose delivered in 2 Gy (EQD2) for SBRT, with the highest dose on top.

Five RCTs^9,10,20,29,33^ reported on complete pain response, and from 1 RCT^8^ these numbers were retrieved from the original data set (all defined according to the ICPRE). The ITT meta-analysis showed that SBRT achieved a higher complete pain response than cEBRT after 1 (RR, 1.43; 95% CI, 1.02-2.01), 3 (RR, 1.80; 95% CI, 1.16-2.78), and 6 (RR, 2.47; 95% CI, 1.24-4.91) months (Figure 3). Also in the PP meta-analysis, SBRT was associated with a higher complete pain response than cEBRT at all 3 time points (eFigure 6 in Supplement 1). No association was seen between SBRT EQD2 and complete pain response.

### Quality of Life

Seven RCTs^7,9,19,20,29,34,35^ analyzed quality of life after radiotherapy, using different quality of life questionnaires at different time points (eTable 2 in Supplement 1). Generally, palliative radiotherapy was associated with improved or maintained quality of life and cEBRT and SBRT were associated with comparable patient-reported quality-of-life outcomes.^7,19,29,35^

### Toxic Effects and Pathologic Fractures

Six RCTs^7,8,9,19,20,33^ reported on toxic effect rates after radiotherapy, and none of them found a statistically significant difference between cEBRT and SBRT. The incidence of toxic effects after radiotherapy varied among RCTs (eTable 3 in Supplement 1).

Six RCTs^7,9,19,20,29,36^ recorded the number of fractures at the irradiated site, and none reported a statistically significant difference between cEBRT and SBRT. The incidence of fractures at the irradiated site varied substantially among studies. None of the RCTs reported on the baseline bone lesion quality (eg, blastic or lytic) or extent of the lesion (eTable 3 in Supplement 1).

### Overall Survival

Seven RCTs^7,8,9,19,20,29,33^ reported overall survival, and none found a statistically significant difference between cEBRT and SBRT. Overall survival was comparable among RCTs: Berwouts et al^29^ reported a median overall survival of 8 (95% CI, 3.6-12.4) months, Nguyen et al^7^ a median of 6.7 (95% CI, 4.6-10.9) months, and Sprave et al^33^ a mean of 7.9 months (SD not reported). The overall 3-month survival was 84% in the trial by Pielkenrood et al^8^ and in the trial by Mercier et al,^20^ it was 88% after cEBRT and 76% after SBRT. Sahgal et al^9^ found that 73% of patients were alive at 6 months after cEBRT and 77% after SBRT. Ryu et al^19^ reported an overall survival of 32% for both cEBRT and SBRT after 2 years.

## Discussion

This systematic review and meta-analysis of 18 studies,^7,8,9,10,11,12,13,19,20,28,29,30,31,32,33,34,35,36^ including 8 RCTs,^7,8,9,10,20,29,33^ found that overall pain response did not differ between patients treated for painful bone metastases with cEBRT or SBRT after 1, 3, or 6 months, but complete pain response was significantly higher after SBRT at all time points. The pooled overall pain response was approximately 52% after cEBRT and approximately 62% after SBRT in the PP population.

Patients with a high performance status or high pain scores at baseline have a higher probability of pain relief than patients who do not.^38,39^ In 3 RCTs,^7,20,29^ patients receiving cEBRT and SBRT had different baseline pain scores, but in only 1 RCT,^20^ the baseline pain scores were higher for patients undergoing SBRT. In the trial by Ryu et al,^19^ patients in the cEBRT group had a statistically significantly higher performance status (90% of patients had a Zubrod status 0-1) compared with the patients in the SBRT group (in which 78% of patients had a Zubrod status 0-1). The differing baseline performance statuses might explain their finding that pain response was higher after cEBRT than after SBRT.

Of 8 included RCTs, 2 RCTs^20,29^ blinded patients for the treatment they received, and 1 RCT^8^ only blinded patients in the cEBRT group. In none of these 3 RCTs, patients experienced a higher overall pain response after SBRT than after cEBRT; however, patients did in most of the 5 unblinded RCTs, which could be due to disappointment bias. Disappointment bias is observed among patients randomized to the control group while hoping to be randomized to the intervention group. If patients know about a new treatment (eg, SBRT) being available but do not receive this treatment because they are assigned to the control group, they may report a more negative outcome. Trials with subjective outcome measures, such as pain scores, are prone to disappointment bias.^40^

One of the included RCTs,^9^ in which SBRT was delivered in 2 fractions of 12 Gy, showed consistently superior (complete) pain response of SBRT over cEBRT. Possibly, fractionation does matter.^41^ A number of possible radiobiological explanations for fractionation exist, including overcoming hypoxia, allowing damage repair by normal tissue cells, and redistribution of cycling cells.^42^ It is possible that this RCT^9^ chose the appropriate SBRT dose regimen with the optimal number of fractions. Another explanation for the consistently superior complete pain response of SBRT in the trial by Sahgal et al^9^ could be the inclusion of a higher proportion of patients with radioresistant tumors (eg, renal cell cancer metastases). SBRT delivered in high doses per fraction may be particularly effective in the treatment of metastases from radioresistant tumors.^43^ In the trial by Sahgal et al,^9^ 26% of patients had metastases from a radioresistant tumor, while the proportion of patients with radioresistant tumors in the other RCTs was less than 10%. Patients with radioresistant tumors may comprise a subgroup for whom SBRT is more effective than cEBRT in relieving pain.

Although the overall pain response did not differ between patients treated with cEBRT and SBRT, the complete pain response was significantly higher for SBRT after 1, 3, and 6 months. Radiotherapy is considered to relieve metastatic bone pain by primarily targeting the biological pathway instead of the mechanical pathway. The mechanical pathway causes pain by directly stimulating afferent pain nerves, and the biological pathway causes pain through a complex process of inflammatory factors present in the microenvironment of bone metastases.^44^ SBRT’s higher local ablative dose may be more successful than cEBRT in completely relieving pain for patients where the biological pathway is mainly causing the metastatic pain. Another possibility is that only RCTs that found a difference in complete pain response reported this outcome.

### Limitations

Our systematic review and meta-analysis has to be interpreted in light of its strengths and limitations. First, this systematic review is strengthened by including unpublished results from an already published RCT,^8^ the full results from a new RCT,^20^ and the recently published largest RCT,^19^ to our knowledge. Second, some previous reviews^14,16^ used odds ratios to compare cEBRT with SBRT instead of RRs. For clinicians, RRs are more intuitive to interpret and, for RCTs, the preferred measure to use unless the outcome is relatively rare.^45^ Our current meta-analysis may be limited by the heterogeneity of the RCTs regarding the dose regimens used for cEBRT and SBRT. For cEBRT, a large meta-analysis^46^ found that single fraction and multiple fraction were associated with similar pain response. For SBRT, the effect of variable dose regimens on pain response remains to be investigated, although no association was observed between EQD2 and pain response. Second, not all RCTs assessed complete pain response at all time points. Third, 1 of the included RCTs^10^ was considered to be at high overall risk of bias, though excluding this study from the meta-analyses did not change the study findings. Fourth, we pooled all RCTs, including spinal and nonspinal lesions, which may have disguised regimens successful in relieving bone pain for specific anatomic localizations. However, since spinal metastases are similar to nonspine osseous metastases in terms of bone involvement and pain relief after standard radiotherapy,^47,48^ the response after SBRT in spinal and nonspine osseous metastases is likely to be similar as well. Fifth, only 1 RCT^9^ reported on the presence of a mechanical component, such as the Spinal Instability Neoplastic Score^49^ or Mirels score.^50^

An individual patient data meta-analysis offers numerous advantages compared with the use of summary data, including enhancement of data quality, enabling different forms of outcomes to be combined, and increased precision of statistical techniques.^51^ We therefore aim to conduct an individual patient data meta-analysis of at least the trials conducted in Belgium^20,29^ and the Netherlands^8,52^ to identify subgroups who benefit from SBRT.

## Conclusions

In this systematic review and meta-analysis of 18 studies,^7,8,9,10,11,12,13,19,20,28,29,30,31,32,33,34,35,36^ including 8 RCTs,^7,8,9,10,20,29,33^ patients with painful bone metastases had a similar overall pain response after SBRT compared with cEBRT, but more patients experienced complete pain response after SBRT. Included RCTs were heterogeneous regarding dose regimens and primary tumors. A more detailed analysis with individual patient data is needed to study the associations of specific dose regimens and could be used to help identifying what subgroups benefit from SBRT.

## Notes

Supplement 1. **eTable 1.** Search Strategy **eTable 2.** Overview of Quality-of-Life Outcomes for the 15 Included Studies **eTable 3.** Overview of Local Control, Toxic Effects, and Progression of Fractures for the 15 Included Studies **eFigure 1.** Flowchart Illustrating the Searches and Screening **eFigure 2.** Separate Funnel Plots of the 8 Included Randomized Clinical Trials **eFigure 3.** Risk of Bias Assessment According to the Revised Cochrane Risk of Bias Tool for the 8 Included Randomized Clinical Trials and According to the Checklist of Strengthening the Reporting of Observational Studies in Epidemiology (STROBE) for the 7 Cohort Studies **eFigure 4.** Pooled Complete Pain Response Among the Per-Protocol Population of the 6 Included Randomized Clinical Trials **eFigure 5.** Meta-Analysis Among Per-Protocol Population on Overall Pain Response at 1, 3, and 6 Months After Radiotherapy of 8 Included Randomized Clinical Trials **eFigure 6.** Meta-Analysis Among Per-Protocol Population on Complete Pain Response at 1, 3, and 6 Months After Radiotherapy of the 6 Included Randomized Clinical Trials **eFigure 7.** Sensitivity Meta-Analyses Among Intention-to-Treat Population on Overall Pain Response and Complete Pain Response at 3 Months After Radiotherapy for 6 Included Randomized Clinical Trials Not at High Risk of Overall Bias

Supplement 2.
Data Sharing Statement
