# Supplementary material for: Stereotactic Body and Conventional Radiotherapy for Painful Bone Metastases: A Systematic Review and Meta-Analysis
Source: JAMA Netw Open. 2024 Feb 12;7(2):e2355409. doi: 10.1001/jamanetworkopen.2023.55409 (PMC10862159; doi:10.1001/jamanetworkopen.2023.55409)
Supplement: Supplement 1. — eTable 1. Search Strategy eTable 2. Overview of Quality-of-Life Outcomes for the 15 Included Studies eTable 3. Overview of Local Control, Toxic Effects, and Progression of Fractures for the 15 Included Studies eFigure 1. Flowchart Illustrating the Searches and Screening eFigure 2. Separate Funnel Plots of the 8 Included Randomized Clinical Trials eFigure 3. Risk of Bias Assessment According to the Revised Cochrane Risk of Bias Tool for the 8 Included Randomized Clinical Trials and According to the Checklist of Strengthening the Reporting of Observational Studies in Epidemiology (STROBE) for the 7 Cohort Studies eFigure 4. Pooled Complete Pain Response Among the Per-Protocol Population of the 6 Included Randomized Clinical Trials eFigure 5. Meta-Analysis Among Per-Protocol Population on Overall Pain Response at 1, 3, and 6 Months After Radiotherapy of 8 Included Randomized Clinical Trials eFigure 6. Meta-Analysis Among Per-Protocol Population on Complete Pain Response at 1, 3, and 6 Months After Radiotherapy of the 6 Included Randomized Clinical Trials eFigure 7. Sensitivity Meta-Analyses Among Intention-to-Treat Population on Overall Pain Response and Complete Pain Response at 3 Months After Radiotherapy for 6 Included Randomized Clinical Trials Not at High Risk of Overall Bias [file jamanetwopen-e2355409-s001.pdf]

Supplemental Online Content

Bindels BJ, Mercier C, Gal R, et al. Stereotactic body and conventional radiotherapy for painful bone metastases: a systematic review and meta-analysis. *JAMA Netw Open*. 2024;7(2):e2355409. doi:10.1001/jamanetworkopen.2023.55409

eTable 1. Search Strategy

eTable 2. Overview of Quality-of-Life Outcomes for the 15 Included Studies

eTable 3. Overview of Local Control, Toxic Effects, and Progression of Fractures for the 15 Included Studies

eFigure 1. Flowchart Illustrating the Searches and Screening

eFigure 2. Separate Funnel Plots of the 8 Included Randomized Clinical Trials

eFigure 3. Risk of Bias Assessment According to the Revised Cochrane Risk of Bias Tool for the 8 Included Randomized Clinical Trials and According to the Checklist of Strengthening the Reporting of Observational Studies in Epidemiology (STROBE) for the 7 Cohort Studies

eFigure 4. Pooled Complete Pain Response Among the Per-Protocol Population of the 6 Included Randomized Clinical Trials

eFigure 5. Meta-Analysis Among Per-Protocol Population on Overall Pain Response at 1, 3, and 6 Months After Radiotherapy of 8 Included Randomized Clinical Trials

eFigure 6. Meta-Analysis Among Per-Protocol Population on Complete Pain Response at 1, 3, and 6 Months After Radiotherapy of the 6 Included Randomized Clinical Trials

eFigure 7. Sensitivity Meta-Analyses Among Intention-to-Treat Population on Overall Pain Response and Complete Pain Response at 3 Months After Radiotherapy for 6 Included Randomized Clinical Trials Not at High Risk of Overall Bias

This supplemental material has been provided by the authors to give readers additional information about their work.

© 2024 Bindels BJ et al. *JAMA Network Open.*

eTable1. Search strategy

"bone and bones" OR bone OR bones OR bony OR skeletal OR osseous OR spine OR spinal
AND
metastasis OR metastases OR metastatic OR neoplasmata OR neoplasm OR neoplasms OR cancer OR cancers OR carcinoma OR carcinomas OR tumor OR tumors OR tumour OR tumours
AND
radiosurgery OR "stereotactic body radiotherapy" OR "stereotactic body radiation therapy" OR "stereotactic body radiosurgery" OR "stereotactic radiosurgery" OR "stereotactic spinal radiotherapy" OR "stereotactic spinal radiosurgery" OR stereotaxis OR sbrt OR srs OR sbrs OR ssr OR sabr OR "stereotactic ablative"

© 2024 Bindels BJ et al. *JAMA Network Open.*

eTable 2. Overview of quality of life outcomes for the 15 included studies[7-13,19,20,28-33] reporting on pain response after conventional external beam radiation therapy compared with stereotactic body radiotherapy for patients with painful bone metastases. Studies were sorted based on years of treatment, with the most recent studies on top.

First author, year	ITT population, No. (cEBRT/SBRT)	Time points for QoL	QoL questionnaires administered	Description of overall outcome QoL	Description of QoL comparison of cEBRT vs SBRT
**Randomized controlled trial**					
Mercier et al,[20] 2023	63/63	3 months	BM-22	Stable or improved QoL scores during follow-up	No significant differences between treatment arms (P values not reported)
Sakr et al,[10] 2020	12/10	NR	-	-	-
Sahgal et al,[9] 2021	115/114	1/3/6 months	QLQ-C30 and BM-22	NR	Financial burden in favor of SBRT (P = 0.03; QLQ-C30); other aspects no significant differences between treatment arms (all P values > 0.05)
Pielkenrood et al,[8] 2021	55/55	1/2/3 months	QLQ-C15-PAL and BM-22[a]	Improved QoL scores in the majority of patients at some point during follow-up[a]	Functional interference (P value not reported; BM-22) and emotional functioning (P value not reported; QLQ-C15-PAL) in favor of cEBRT; other aspects no significant differences between treatment arms (P values not reported)[a]
Nguyen et al,[7] 2019	79/81	1-3/6/9/12 months	MDASI	Improved QoL scores during follow-up	No significant differences between treatment arms (P values not reported)
Sprave et al,[33] 2018	30/30	3/6 months	QLQ-BM-22, QLQ-FA13 and QSC-R10[b]	NR[b]	No significant differences between treatment arms (All P values > 0.05)[b]
Ryu et al,[19] 2023	136/217	NR	FACT-G and EQ-5D	Stable or improved QoL scores during follow-up	No significant differences between treatment arms (P values not reported)
Berwouts et al,[29] 2015	15/15	2 weeks, 1 month	QLQ-C15-PAL and BM-22	Stable or improved QoL scores during follow-up	No significant differences between treatment arms (All P values > 0.05)
**Cohort studies**					
Ito et al,[11] 2022	81/81	NR	-	-	-
Marvaso et al,[12] 2022	59/62	NR	-	-	-
Van de Ven et al,[13] 2020	66/65	2 weeks, 1/2/3/6/12 months	BM-22, QLQ-C15-PAL and EQ-5D	Stable QoL scores during follow-up	Physical functioning in favor of SBRT (P = 0.04; QLQ-C15-PAL); other aspects no significant differences between treatment arms (P values not reported)
Amini et al,[28] 2015	45/50	NR	-	-	-
Sohn et al,[32] 2016	28/28	NR	-	-	-
Sohn et al,[31] 2014	13/13	NR	-	-	-
Haley et al,[30] 2011	22/22	NR	-	-	-

[a]Quality of life data was retrieved from a secondary analysis study,[34] [b]Quality of life data was retrieved from a secondary analysis study.[35] Abbreviations: cEBRT; conventional external beam radiotherapy, NR; not reported, QoL; Quality of life, SBRT; stereotactic body radiotherapy

© 2024 Bindels BJ et al. *JAMA Network Open.*

eTable 3. Overview of local control, toxicity, and (progression of) fractures for the 15 included studies[7-13,19,20,28-33] reporting on pain response after conventional external beam radiation therapy compared with stereotactic body radiotherapy for patients with painful bone metastases. Studies were sorted based on years of treatment, with the most recent studies on top.

First author, year	ITT population, No. (cEBRT/SBRT)[a]	Time points for LC/LPFS	LC/LPFS rates for cEBRT	LC/LPFS rates for SBRT	Patients with toxicity after cEBRT[a], No. (%)	Patients with toxicity after SBRT[a], No. (%)	Patients with any (progression of) fractures at irradiated site after cEBRT, No. (%)	Patients with any (progression of) fractures at irradiated site after SBRT, No. (%)
**Randomized controlled trials**								
Mercier et al,[20] 2023	63/63	NR	NR	NR	1/63 (1.6%)	1/63 (1.6%)	1/63 (1.6%)	1/63 (1.6%)
Sakr et al,[10] 2020	12/10	NR	-	-	NR	NR	NR	NR
Sahgal et al,[9] 2021	115/114	LPFS 6 months	69.0%	75.0%	8/115 (7.0%)	7/110 (6.4%)	20/115 (17.4%)	12/110 (10.9%)
Pielkenrood et al,[8] 2021	55/55	NR	-	-	0/44 (0.0%)	0/45 (0.0%)	NR	NR
Nguyen et al,[7] 2019	79/81	LC 1 year<br>LC 2 years	94.1%<br>90.3%	100%<br>100%	10/79 (12.6%)	9/81 (11.1%)	0/79 (0.0%)	1/81 (1.2%)
Sprave et al,[33] 2018	30/30	NR	-	-	0/28 (0.0%)	0/27 (0.0%)	2/28 (7.1%)[b]	7/27 (25.9%)[b]
Ryu et al,[19] 2023	136/217	NR	-	-	10/117 (8.5%)	39/202 (19.3%)	25/130 (19.2%)	39/209 (18.6%)
Berwouts et al,[29] 2015	15/15	NR	-	-	NR	NR	1/15 (6.7%)	1/15 (6.7%)
**Cohort studies**								
Ito et al,[11] 2022	81/81	LC 1 year	66.7%	89.8%	3/81 (3.7%)	2/81 (2.5%)	1/81 (1.2%)	2/81 (2.5%)
Marvaso et al,[12] 2022	59/62	NR	-	-	NR	NR	NR	NR
Van de Ven et al,[13] 2020	66/65	LPFS 1 year	19.0%	54.0%	NR	NR	NR	NR
Amini et al,[28] 2015	45/50	LC 1 year<br>LC 2 years	45.1%<br>22.8%	74.1%<br>61.4%	0/45 (0.0%)	0/50 (0.0%)	NR	NR
Sohn et al,[32] 2016	28/28	LC 6 months<br>LC 1 year	64.0%<br>32.0%	59.0%<br>25.0%	0/28 (0.0%)	0/28 (0.0%)	1/28 (3.6%)	5/28 (17.9%)
Sohn et al,[31] 2014	13/13	LC 6 months<br>LC 1 year	58.3%<br>29.2%	100%<br>85.7%	0/13 (0.0%)	0/13 (0.0%)	0/13 (0.0%)	2/13 (15.4%)
Haley et al,[30] 2011	22/22	NR	-	-	0/22 (0.0%)	0/22 (0.0%)	NR	NR

[a]Toxicity was defined according to Common Toxicity Criteria (only grades ≥ 3),[b]local response data was retrieved from a secondary analysis study,[36] Abbreviations: cEBRT; conventional external beam radiotherapy, ITT; intention-to-treat, LC; local control, LPFS; local progression-free survival, NR; not reported, SBRT; stereotactic body radiotherapy.

© 2024 Bindels BJ et al. *JAMA Network Open.*

Supplementary Figures

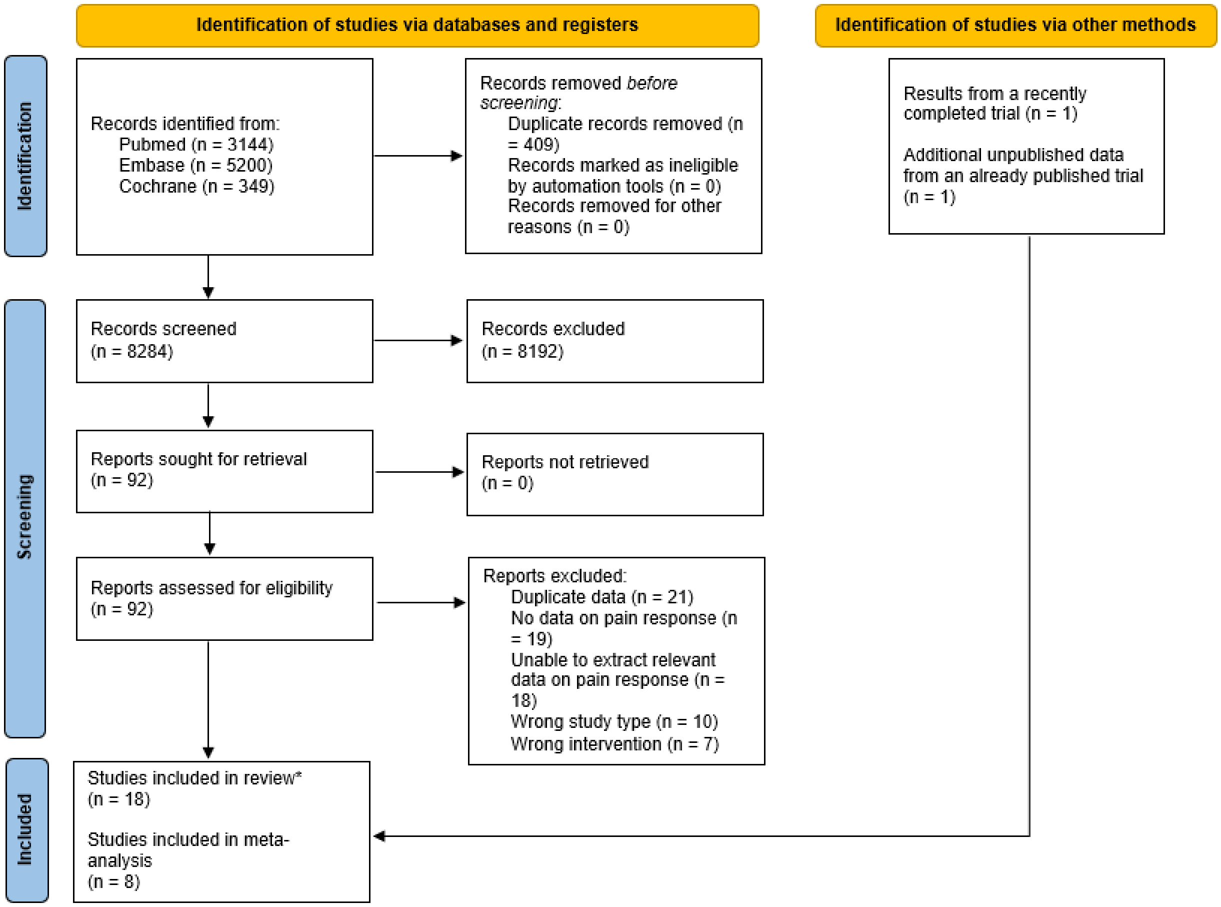

eFigure 1. Flow diagram illustrating the searches, screening. Ultimately, 18 studies[7-13,19,20,28-36] were included in the review and eight studies[7-10,19,20,29,33] were included in the meta-analysis.

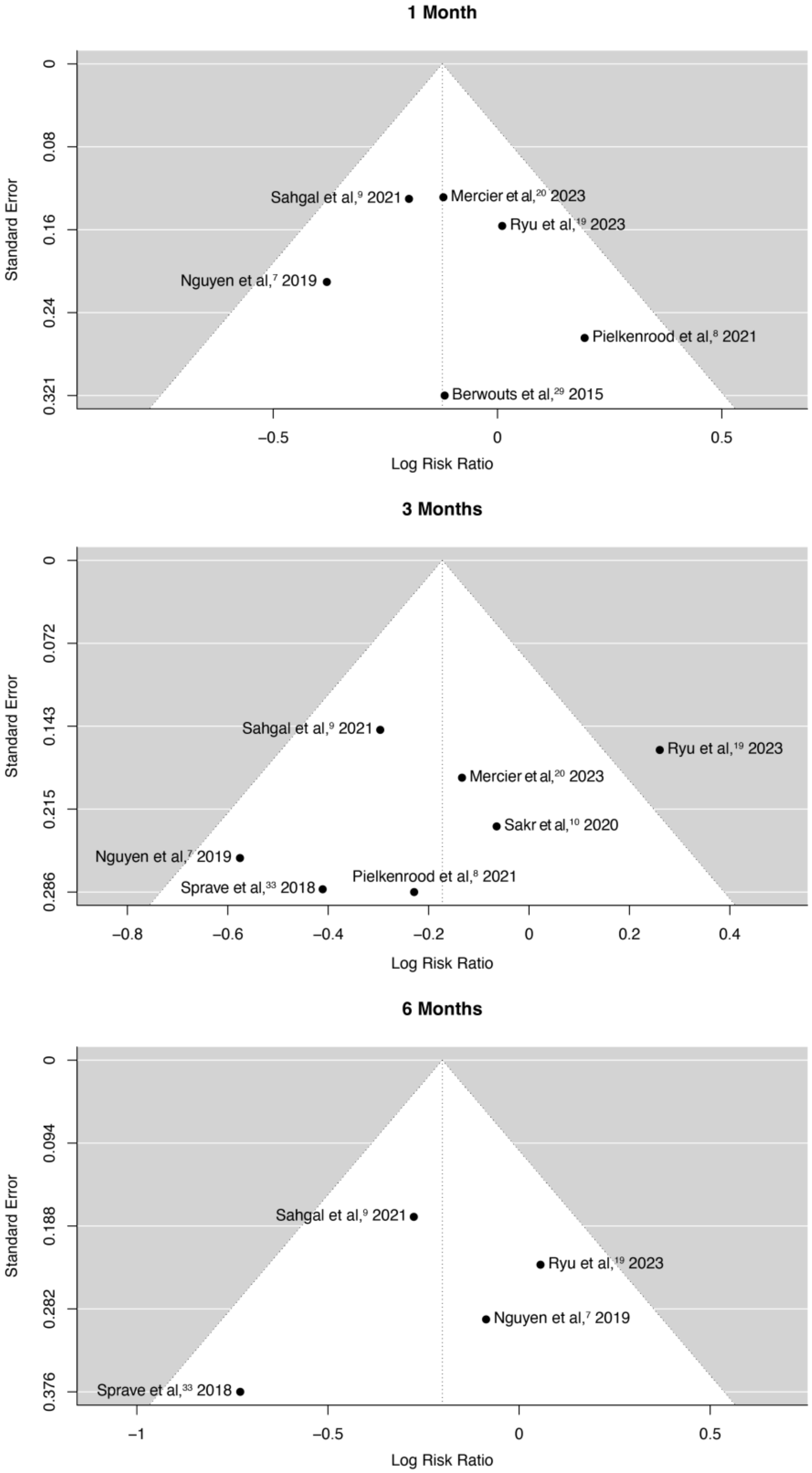

eFigure 2. Separate funnel plots of the eight included randomized controlled trials[7-10,19,20,29,33] evaluating pain response for bone metastases at 1, 3, and 6 months.

© 2024 Bindels BJ et al. *JAMA Network Open.*

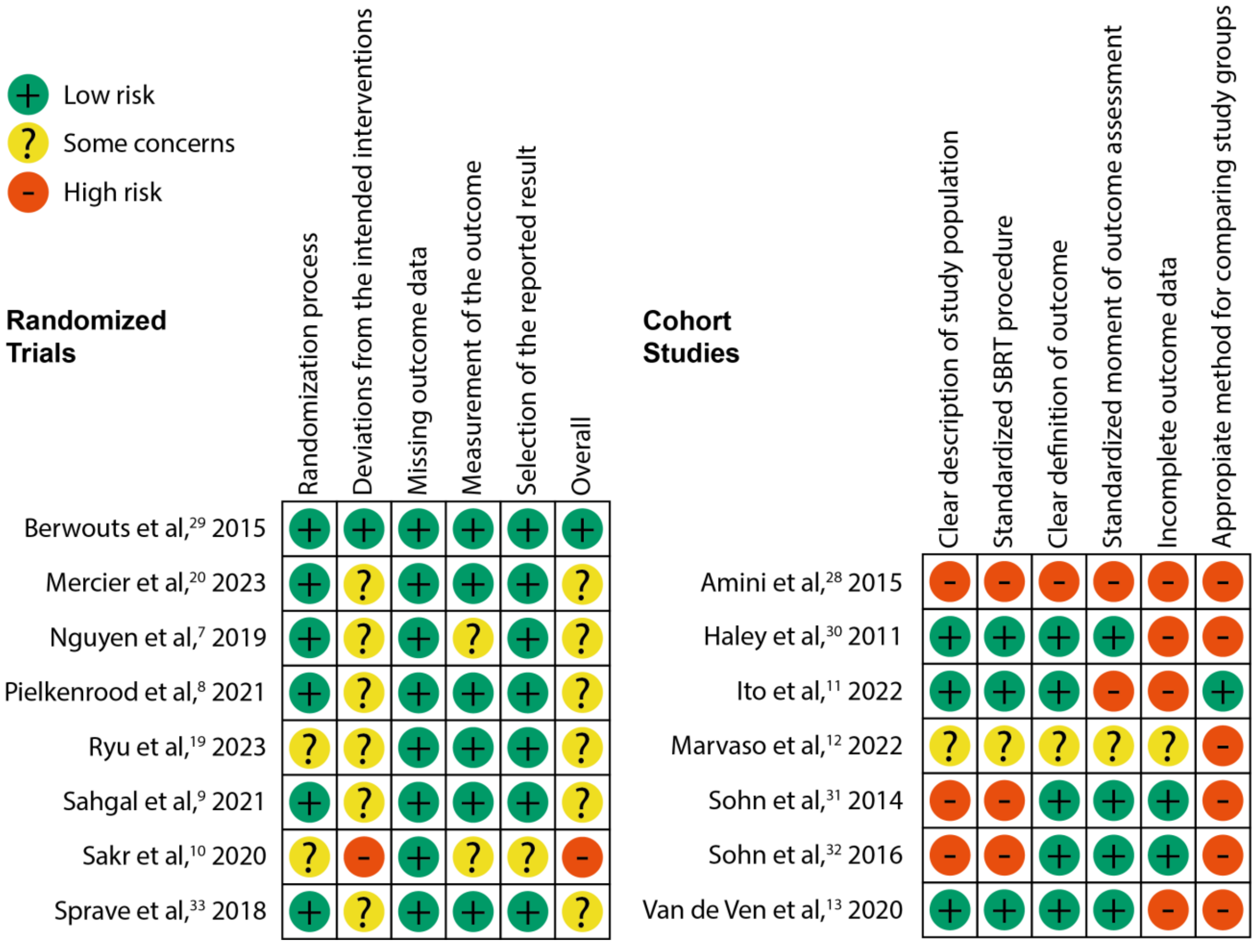

eFigure 3. Risk of bias assessment according to the revised Cochrane Risk of Bias tool (RoB2) for the eight included randomized controlled trials[7-10,19,20,29,33] and according to the checklist of Strengthening the Reporting of Observational studies in Epidemiology (STROBE) for the seven cohort studies[11-13,28,30-32] reporting on pain response after conventional external beam radiation therapy compared with stereotactic body radiotherapy for patients with painful bone metastases.

© 2024 Bindels BJ et al. *JAMA Network Open.*

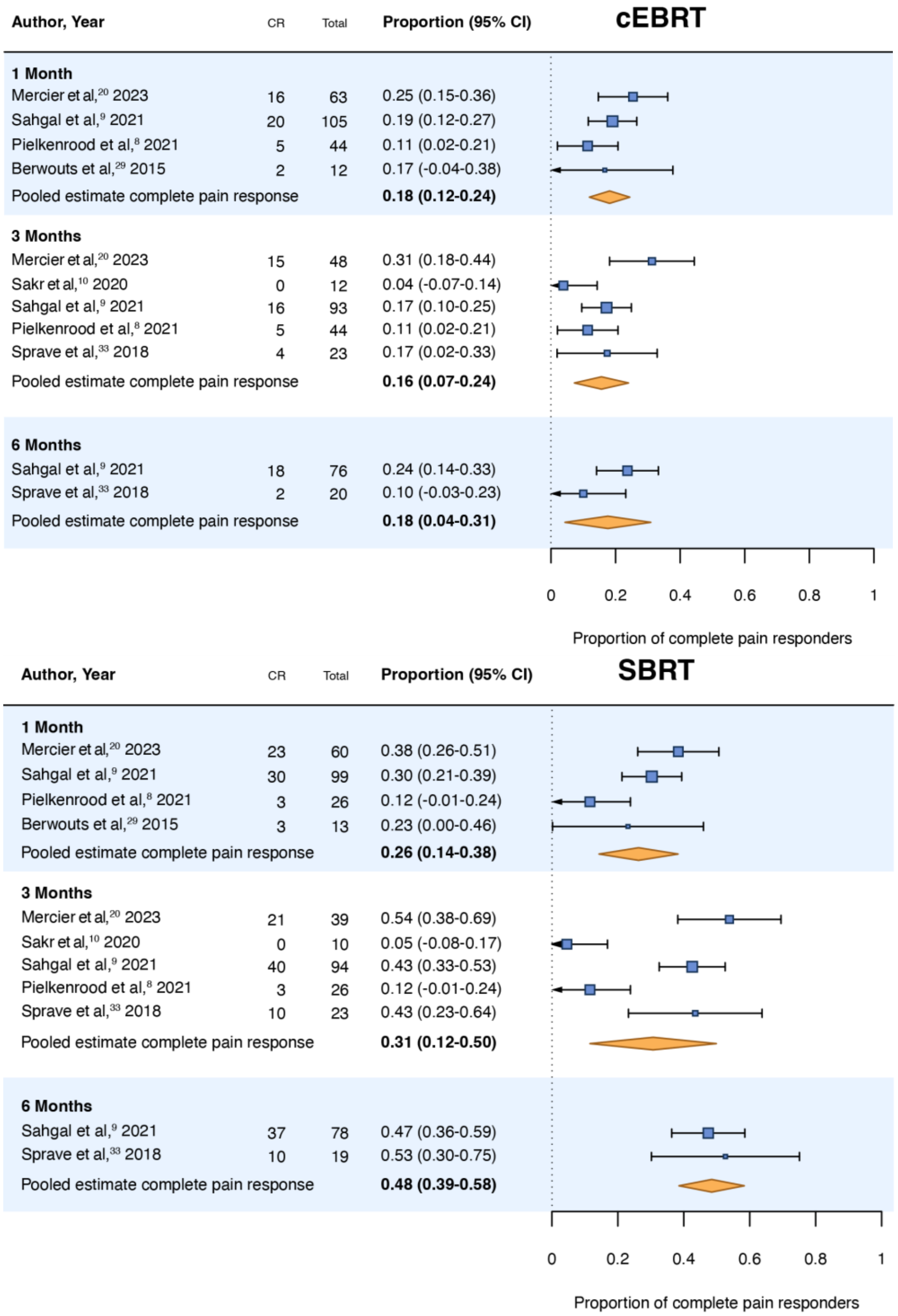

e**Figure 4.** Pooled complete pain response (CR) among the per-protocol population of the six included randomized controlled trials[8-10,20,29,33] at 1, 3 and 6 months after conventional external beam radiotherapy (cEBRT) and stereotactic body radiotherapy (SBRT). Studies were sorted based on years of treatment, with the most recent studies on top. Because the raw proportions were used, some calculated confidence intervals have negative numbers.

© 2024 Bindels BJ et al. *JAMA Network Open.*

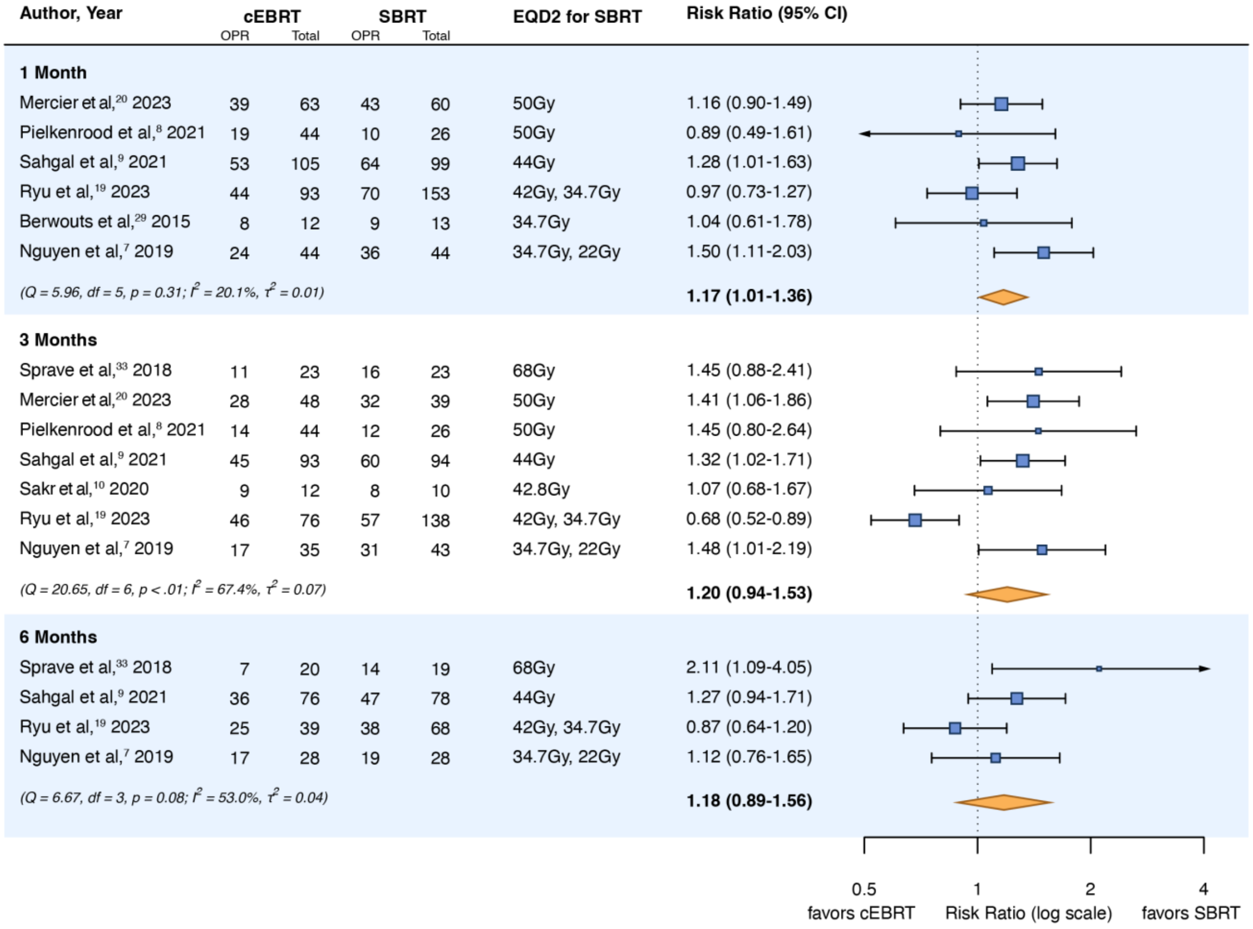

eFigure 5. Meta-analysis among per-protocol population on overall pain response (OPR) at 1, 3, and 6 months after radiotherapy of eight included randomized trials[7-10,19,20,29,33] comparing conventional external beam radiotherapy (cEBRT) with stereotactic body radiotherapy (SBRT). Studies were sorted based on the equivalent dose delivered in 2Gy (EQD2) for SBRT, with the highest dose on top.

© 2024 Bindels BJ et al. *JAMA Network Open.*

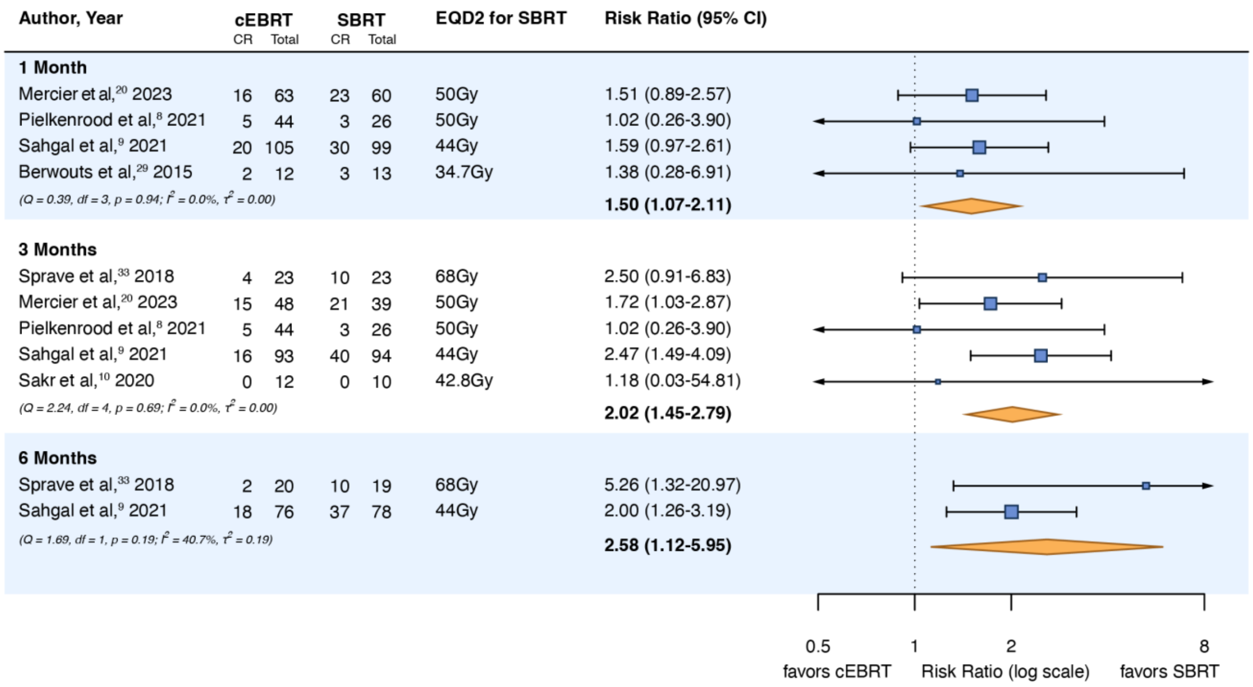

eFigure 6. Meta-analysis among per-protocol population on complete pain response (CR) at 1, 3, and 6 months after radiotherapy of the six included randomized trials[8-10,20,29,33] comparing conventional external beam radiotherapy (cEBRT) with stereotactic body radiotherapy (SBRT). Studies were sorted based on the equivalent dose delivered in 2Gy (EQD2) for SBRT, with the highest dose on top.

© 2024 Bindels BJ et al. *JAMA Network Open.*

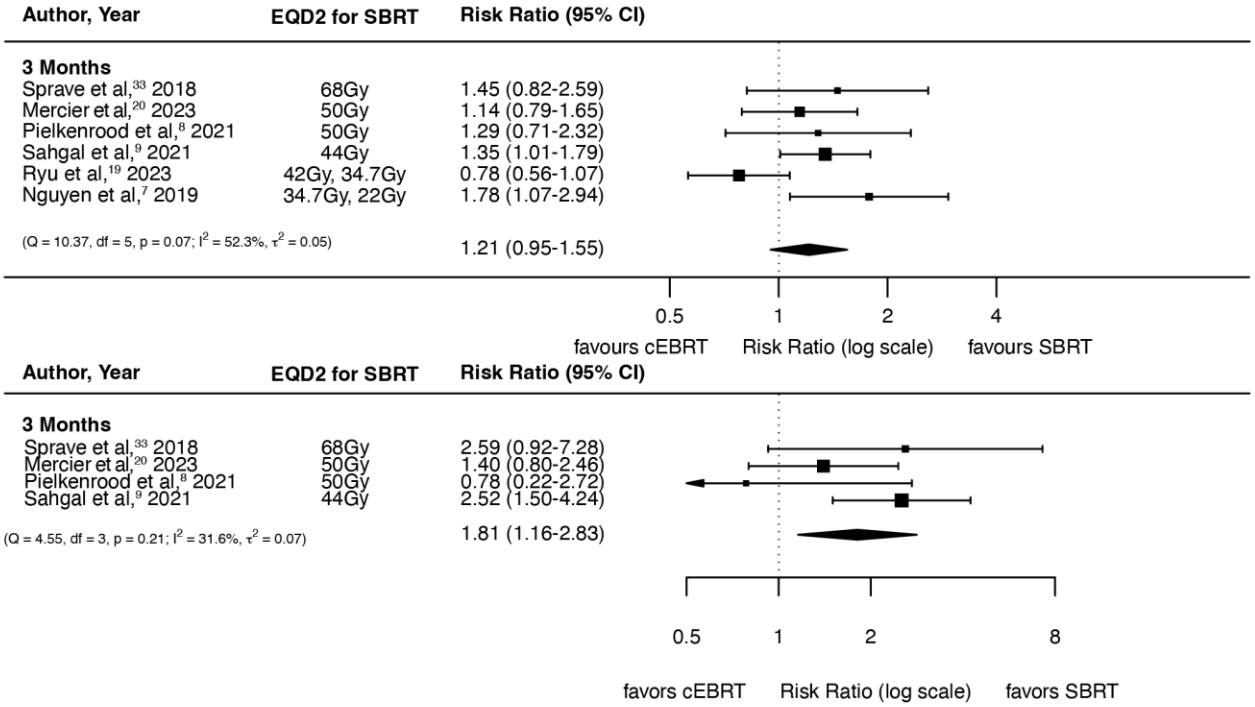

eFigure 7. Sensitivity meta-analyses among intention-to-treat population on overall pain response (above) and complete pain response (below) at 3 months after radiotherapy for six included randomized trials[7,8,9,19,20,33] not at high risk of overall bias. The trials compared conventional external beam radiotherapy (cEBRT) with stereotactic body radiotherapy (SBRT). Studies were sorted based on the equivalent dose delivered in 2Gy (EQD2) for SBRT, with the highest dose on top.

© 2024 Bindels BJ et al. *JAMA Network Open.*
