# Supplementary material for: Stereotactic Body and Conventional Radiotherapy for Painful Bone Metastases: A Systematic Review and Meta-Analysis
Source: JAMA Netw Open. 2024 Feb 12;7(2):e2355409. doi: 10.1001/jamanetworkopen.2023.55409 (PMC10862159; doi:10.1001/jamanetworkopen.2023.55409)
Supplement: Supplement 2. — Data Sharing Statement [file jamanetwopen-e2355409-s002.pdf]

Data Sharing Statement

Bindels. Stereotactic Body and Conventional Radiotherapy for Painful Bone Metastases. *JAMA Netw Open*. Published February 12, 2024. doi:10.1001/jamanetworkopen.2023.55409

Data
Data available: No

Additional Information
Explanation for why data not available: No individual patient data available.
